# The impact of position-orientation adaptive smoothing in diffusion weighted imaging—From diffusion metrics to fiber tractography

**DOI:** 10.1371/journal.pone.0233474

**Published:** 2020-05-20

**Authors:** Jia Yang, Barbara Carl, Christopher Nimsky, Miriam H. A. Bopp

**Affiliations:** 1 Department of Neurosurgery, University of Marburg, Marburg, Germany; 2 Department of Neurosurgery, The First Affiliated Hospital of Sun Yat-sen University, Guangzhou, China; 3 Department of Neurosurgery, Helios Dr. Horst Schmidt Kliniken, Wiesbaden, Germany; 4 Marburg Center for Mind, Brain and Behavior (MCMBB), Marburg, Germany; University of North Carolina at Chapel Hill, UNITED STATES

## Abstract

In contrast to commonly used approaches to improve data quality in diffusion weighted imaging, position-orientation adaptive smoothing (POAS) provides an edge-preserving post-processing approach. This study aims to investigate its potential and effects on image quality, diffusion metrics, and fiber tractography of the corticospinal tract in relation to non-post-processed and averaged data. 22 healthy volunteers were included in this study. For each volunteer five clinically applicable diffusion weighted imaging data sets were acquired and post-processed by standard averaging and POAS. POAS post-processing led to significantly higher signal-to-noise-ratios (p < 0.001), lower fractional anisotropy across the whole brain (p < 0.05) and reduced intra-subject variability of diffusion weighted imaging signal intensity and fractional anisotropy (p < 0.001, p = 0.006). Fiber tractography of the corticospinal tract resulted in significantly (p = 0.027, p = 0.014) larger tract volumes while fiber density was the lowest. Similarity across tractography results was highest for POAS post-processed data (p < 0.001). POAS post-processing enhances image quality, decreases the intra-subject variability of signal intensity and fractional anisotropy, increases fiber tract volume of the corticospinal tract, and leads to higher reproducibility of tractography results. Thus, POAS post-processing supports a reliable and more accurate fiber tractography of the corticospinal tract, being mandatory for the clinical use.

## Introduction

Diffusion weighted imaging (DWI) in combination with fiber tractography is currently the most commonly used approach to map white matter tracts non-invasively *in-vivo* [1–5] and has also been used to evaluate the changes of microstructural properties in brain disorders [6, 7]. Therefore, DWI gained wide applications in clinical settings, such as fiber tractography and analysis of diffusion metrics [8–12]. Due to the growing interest in DWI and its clinical and scientific applications it is essential to consider factors affecting the quality of data acquisition, diffusion metrics, and fiber tractography.

DWI data quality is affected by acquisition parameters, subject motion, eddy currents, physiological noise, and image distortions [13–16], but also suffers from low spatial resolution and its effects such as blurring and localized signal loss [17]. Influenced by these parameters and effects, the analysis of DWI data can be prone to errors possibly also affecting clinical decisions.

For high quality DWI data analysis high spatial resolution as well as high angular resolution is required [18, 19]. High spatial resolution is achieved by using smaller voxel sizes, e.g. high in-plane resolution (anisotropic voxels) or isotropic reduced voxel volumes, reducing partial volume artifacts. In this way, diffusion properties can be depicted more accurately and thereby also fiber tractography might be improved [19, 20]. Angular resolution affects the reliability of diffusion measures [19, 20] as well as the stability and complexity of fiber tractography [21–23]. For example, high angular resolution diffusion imaging (HARDI) allows for the differentiation of multi fiber populations, overcoming the drawbacks of common diffusion tensor imaging (DTI)-based fiber tractography [23–25]. However, increasing spatial and angular resolution leads to longer acquisition times, not routinely applicable in the clinical setting. Furthermore, increasing spatial resolution or applying larger b-values commonly used in high angular resolution data leads to lower signal to noise ratios (SNR), also resulting in poor image quality and error-prone estimation of diffusion characteristics [26–28].

In diffusion weighted imaging, image quality and SNR are affected by motion, susceptibility-related distortion, physiological effects, and scan duration [28]. The SNR can be improved by using an averaging approach requiring multiple repeated image sets [29] with increased acquisition time, or by the application of post-processing approaches based on a standard data set. As especially in the clinical setting acquisition time is limited due to the patient’s condition, post-processing approaches provide an opportunity to improve image quality.

So far, several denoising approaches have been proposed to enhance the image quality and the SNR, such as Gaussian filtering [30], wavelet transformation [31], Perona-Malik filter [32], non-linear anisotropic diffusion filter [33], the propagation-separation approach [34], non-local means [35], linear minimum mean square error estimation [36], local principal component analysis [37], random matrix theory [38] and higher-order singular value decomposition [39]. Some advanced ones specifically focusing on diffusion weighted data and addressing image specific features such as Rician or non-central Chi noise distribution applying advanced and adapted methods of non-local means based approaches [40–43] or even machine learning approaches such as dictionary learning [44].

Position-orientation adaptive smoothing (POAS) works based on a propagation-separation approach [34, 45], yielding an iterative adaptive multi-scale approach for smoothing in both, voxel and diffusion gradient space [46, 47]. This smoothing approach can immediately be applied to diffusion weighted image data prior to any kind of modeling of diffusion properties and thereby directly smooth diffusion weighted image data acquired on a single q-shell, as clinically most routinely used. In this way especially no model-specific bias is integrated in the data and any diffusion model available can be used after the application of POAS. For its application several parameters have to be chosen such as the number of iterations, the adaptation, the noise level, the amount of initial smoothing on the sphere of diffusion weighting directions as well as the parallel imaging factor. As a result of its adaptive properties this approach is capable of reducing noise in diffusion weighted image while avoiding blurring of inherent fine anisotropic structures, is reported to be edge preserving and to preserve discontinuities, which is quite important for the preservation of accuracy and precision in estimation of diffusion properties and fiber tractography [46, 47]. For further details on the POAS algorithm, see [46].

In this study, we investigated the contribution of POAS post-processing on the improvement of image quality and SNR, diffusion characteristics and fiber tractography in comparison to the original data and the averaging approach with longer acquisition times. Therefore, at first, image quality of DWI data (using SNR) was evaluated. Second, the effect on diffusion metrics such as fractional anisotropy (FA), mean diffusivity (MD) and intra-subject variability of DWI signal and FA value is analyzed. Finally, we evaluated the effect of POAS post-processing on fiber tractography of the corticospinal tract (CST).

## Materials and methods

### Volunteers

22 healthy subjects (mean age: 27.45 ± 6.10 years, male / female ratio: 9 / 13) were included in this study to evaluate the impact of POAS post-processing on DWI image quality and associated measures in contrast to standard imaging and image averaging. MRI data of all subjects were acquired during a single session. All subjects were right-handed. None of the subjects had any confirmed organic brain diseases, any severe medical and/or neurological condition of lifetime substance dependence.

Written informed consent was provided by all subjects after complete description of the study procedures (experimental setting, mechanism and risks of MRI data acquisition). The study protocol was approved by the local ethics committee at the University of Marburg according to the declaration of Helsinki (reference number 162/12).

### MRI Data acquisition

All MRI data sets were acquired at a 3T MRI System (Tim Trio, Siemens, Erlangen, Germany) using a 12-channel head matrix Rx-coil. Data acquisition included a T1-weighted magnetization prepared rapid gradient echo (MPRAGE) sequence and five repeated DWI data sets with identical parameters, using a single-shot echo planar imaging sequence with parameters as follows:

T1-MPRAGE: repetition time (TR) 1900 ms, echo time (TE) 2.26 ms, inversion time (TI) 900 ms, Field of View (FoV) 256 mm, matrix 256 x 256, slice thickness (ST) 1 mm, distance factor 50%, flip angle 9°, 176 slices, parallel imaging (GRAPPA) with factor 2.

DWI: TR 7800 ms, TE 90 ms, FoV 256 mm, matrix 128 x 128, ST 2 mm distance factor 0%, 40 slices, GRAPPA with factor 2, 30 diffusion encoding gradients, high-b-value 1000 s/mm^2^, seven intermitting low-b-value (0 s/mm^2^) images, axial slices, phase encoding direction anterior >> posterior, resulting voxel size: 2x2x2 mm^3^.

Data acquisition took about 30 minutes per subject.

The image volume of the DWI data sets was aligned in parallel to the connecting line of anterior and posterior commissure (sagittal section) and in parallel to the midsagittal plane, covering the entire cerebrum. Each data set was visually inspected. If visual inspection showed at least one volume with severe artefacts the subject would have been excluded. However, in this study, no severe artefacts or alterations were seen.

The subjects were positioned with head first and in a supine position in the MRI scanner in a dimmed environment. Subjects’ arms were positioned comfortably beside the body. The subject’s head was positioned in prolongation of the body-line and fixated with soft foam rubber pads to minimize head movements and standardize head position, with the nasal bone at the isocenter of the magnetic field.

### Data analysis

The effect of POAS post-processing was evaluated at different levels of processing from DWI data and diffusion tensor metrics to fiber tractography and reliability. For each volunteer the acquired five original data sets, the five POAS post-processed data sets as well as an averaged data set out of the original data sets were considered.

#### Preprocessing

First, all acquired data sets were corrected for head bulk motion and eddy currents using “eddy”, a tool for correction of eddy currents and subject motion in diffusion data [48] implemented in FSL [49–51] (FMRIB Software Library, Oxford, United Kingdom), using standard parameters. For the average data set the corrected images were linearly aligned to the first data set based on the b0 images and averaged afterwards. POAS post-processing was applied to all corrected image sets using the POAS4SPM toolbox in SPM (statistical parametric mapping) [52, 53], with parameters as follows: coil = 1, sigma = 70, lambda = 12, K* = 12, Kappa = 0.8. All the parameters were chosen based on [52] and recent DWI scanning parameters at the used MRI systems. For each individual DWI data set applying POAS post-processing took about 40min (Windows 10 operation system, Intel(R) Core(TM) i5 CPU, 2.40GHZ, 12GB RAM).

#### Visual inspection and SNR estimation

**Fig 1:**
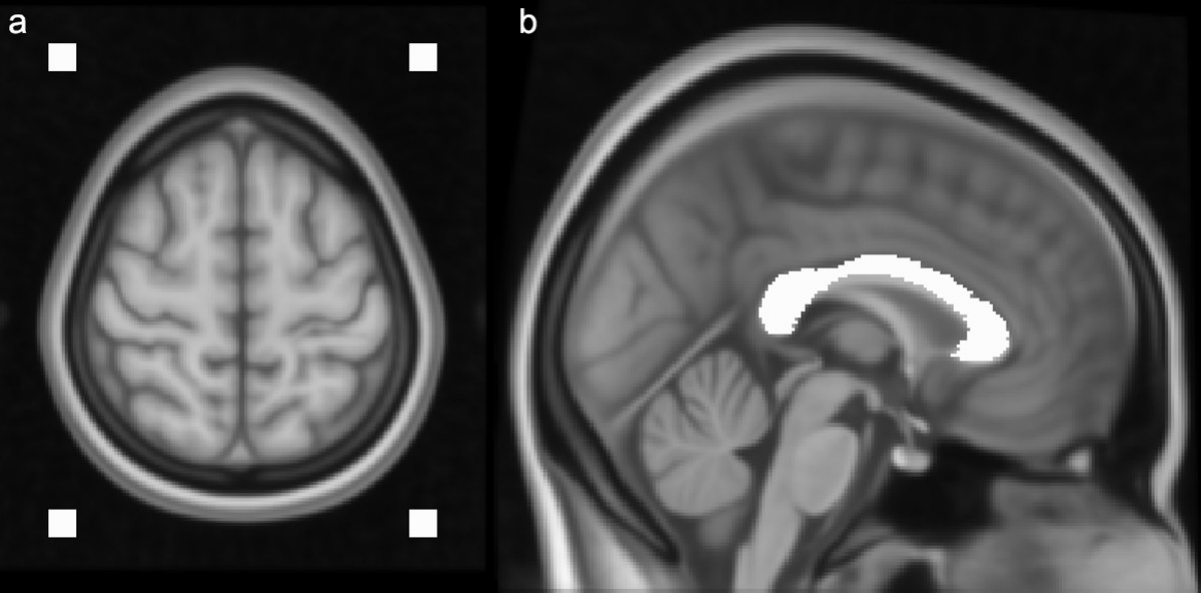
ROI definition for SNR calculation. To calculate the SNR four ROIs within the background of the image were used to estimate image noise (a) as well as a single ROI in a homogeneous region within the brain, in this case the corpus callosum (b).

After correction of head bulk motion and eddy currents and application of averaging or POAS post-processing, all resulting data sets were visually inspected. In addition to visual inspection of image quality, a quantitative analysis was performed using an estimation of the SNR. The same regions of interest (ROIs) were used for estimation of the SNR across all image sets. Therefore, first, all data sets were normalized (transformation to MNI152 standard space). The SNR was calculated as mean signal intensity divided by the standard deviation of noise signal intensity in non-diffusion weighted images. Mean signal intensity was derived using a ROI within the corpus callosum (Fig 1B), standard deviation of noise signal intensity was derived using four ROIs in the image background (Fig 1A).

#### Tract-based spatial statistics (TBSS) analysis of FA and MD

TBSS analysis [54] was conducted to evaluate alterations of FA and MD across the three groups. Therefore, after preprocessing, tensor data were calculated using *dtifit* within the FSL Diffusion Toolbox (FDT). Additionally, all created image sets were normalized to the MNI152 (Montreal Neurological Institute) template using non-linear registration [55] and all transformed datasets were subsequently resampled, resulting in a spatial resolution of 1x1x1 mm^3^. Subsequently, a mean FA-image was created and thinned to generate a mean FA skeleton. Finally, all aligned FA datasets were projected onto the mean FA skeleton using a non-maximum suppression threshold of FA ≥ 0.20. The resulting data are fed into the voxel-wise analysis.

Voxel-wise statistical analysis of differences across the three groups was performed on the whole-brain mean FA skeleton to identify white matter tracts significantly affected. Therefore, a general linear model (GLM), set up using the FSL Randomize Tool [56, 57] using a tripled t-test. All contrasts were analyzed according to permutation-based non-parametric inference with 500 random permutations using threshold-free cluster enhancement (TFCE) [58] to correct for multiple testing. The significance level was set to p < 0.05. To further analyze the effect on other diffusion parameters such as MD, all corresponding MD maps were transformed accordingly, projected on the FA skeleton and processed comparable to the FA data using the same GLM for statistical analysis.

#### Analysis of intra-subject variability in DWI intensity and FA across the five data sets

To further evaluate the intra-subject variability of DWI signal intensity and FA across the five original repeated DWI data sets and FA images as well as the POAS post-processed corresponding data a coefficient of variation (CV) was assessed. First, for each volunteer a standard deviation (SD) map and an average map was calculated for the original and POAS post-processed DWI data and FA images, respectively. Second, the CV maps were created by dividing the SD map by the average map. Finally, to evaluate the intra-subject variability between original and post-processed data using POAS, the CV difference map was considered subtracting the POAS post-processed CV maps from the original CV maps. In addition, for each CV difference map a histogram was calculated.

#### Fiber tractography of the CST and tract profile analysis

The effect of POAS on fiber tractography was evaluated regarding the corticospinal tract (CST). After preprocessing and normalization fiber tractography was performed applying an automated approach using the automated fiber quantification software AFQ (Vista Lab, Stanford University, USA) [59] to reduce manual bias [60, 61]. First, whole brain fiber tractography was conducted applying the deterministic FACT algorithm. Second, the tractography result was segmented into fiber groups, such as the CST, using predefined standard ROIs [60]. The standard ROIs were derived from MNI-JHU-tracts-ROI [59, 62] and transformed accordingly.

Based on the fiber tractography results the tract volume was calculated for each tract based on the number of voxels including fiber tracts and the voxel volume. Furthermore, mean FA and mean MD were calculated as tract profile in AFQ as well as the fiber density by dividing the number of streamlines by tract volume. All those scalar measures (fiber volume, fiber density, mean FA, and mean MD across the CST) were compared among the three groups (original, POAS post-processed, and average data). As standard reference for the CST, additionally a constrained spherical deconvolution (CSD) reconstruction was performed allowing for higher complexity and reliability [63–65].

#### Reproducibility and similarity analysis of the CST

The Jaccard coefficient (JC), also known as “intersection over union measure”, is a normalized measure of overlap typically used to compare sample sets and is defined as the size of the intersection divided by the size of the union of both samples [66, 67] and could reflect the similarity not only in tract volume but also in tract shape. The reproducibility and similarity among the CST reconstructions based on original DWI data and POAS post-processed DWI data was analyzed respectively applying JC. The similarity between both groups was also analyzed using the JC of the CST reconstructions based on original vs. POAS post-processed data. Finally, the similarity analysis between CST reconstructions based on original data and the standard reference as well as between CST reconstructions based on POAS post-processed data and the standard reference was assessed.

### Statistical analysis

Statistical analysis, except for TBSS analysis, was performed in SPSS Statistics 24.0 (IBM, Armonk, USA). Gaussian distribution of the data was evaluated using the Kolmogorov-Smirnov and Shapiro-Wilk test. In the case of Gaussian distribution, a t-test or a randomized block design analysis of variances (ANOVA) was applied to assess significant differences among the groups followed by Tukey HSD post hoc tests to further specify the differences. In case of no Gaussian distribution a Wilcoxon signed-rank test was applied. The significance level was set to p < 0.05.

## Results

### Visual inspection and SNR estimation

**Fig 2:**
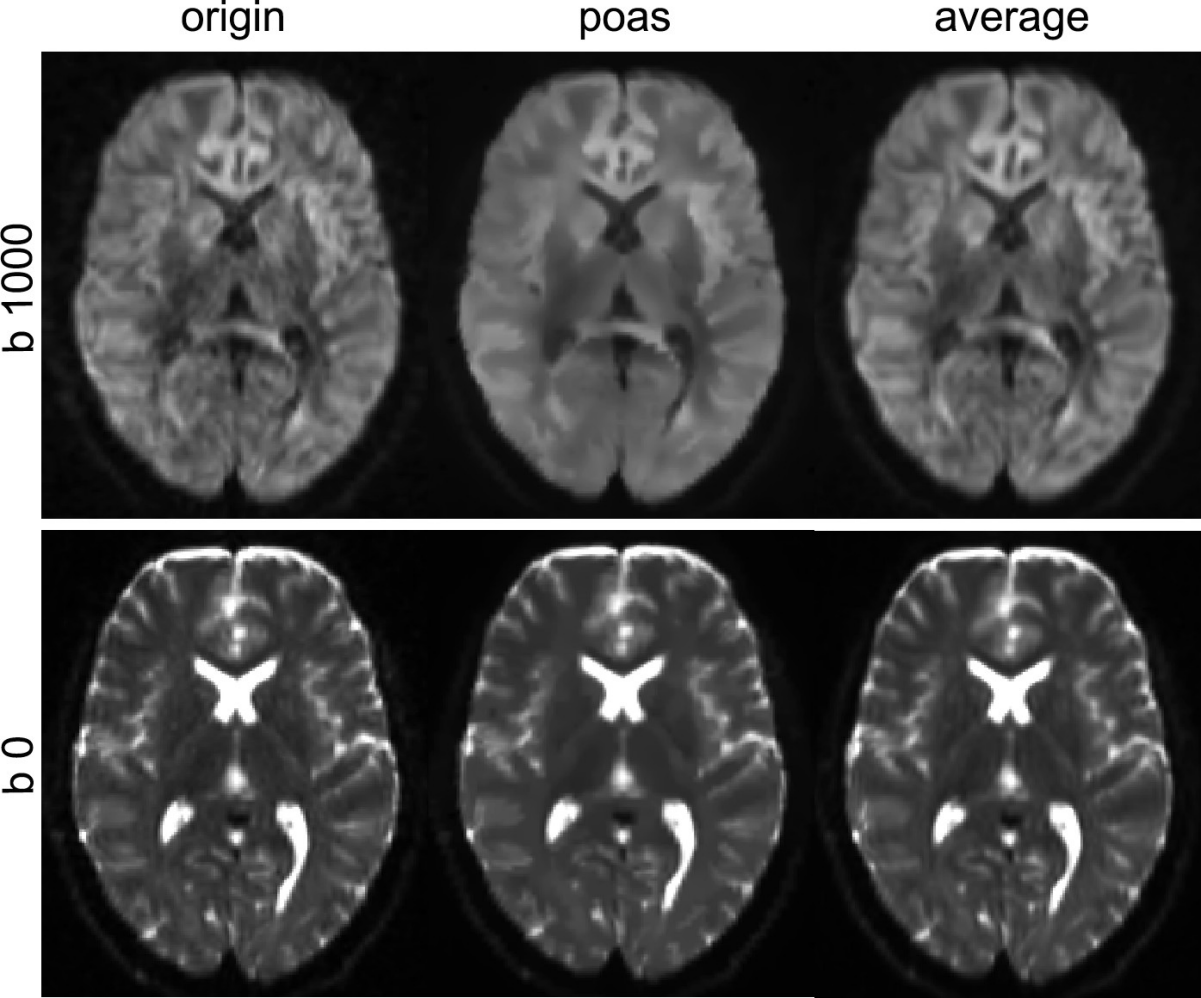
Effect of POAS post-processing and averaging. Visualization of the effect of averaging and POAS post-processing on non-weighted (b = 0 s/mm^2^) and diffusion weighted images (b = 1000 s/mm^2^).

All DWI images including the five original data sets, the five POAS post-processed data sets as well as the averaged original data sets, were visually inspected (J. Y.). While within the b0-images no obvious alterations were found among the different groups, more obvious differences were seen within the high-b-value images. Original data showed increased noisy structures within the brain parenchyma with unclear boundaries between grey and white matter. After averaging, image quality improved with reduced noise, but further blurring of the data. Visual inspection of POAS post-processed data showed further noise reduction in contrast to averaging, with saliently differentiation between gray and white matter. No obvious blurring, as well as no decrease of image intensity was seen. For further illustration of the effect of POAS and averaging in contrast to original DWI data an exemplary comparison of the three groups is shown in Fig 2.

The SNR was estimated quantitatively to compare the image quality among the three groups using the same ROIs for all data sets. Across all subject the mean SNR of the original data was 133.33 ± 32.60, while mean SNR was 178.70 ± 64.59 for averaged data and 218.27 ± 78.09 for POAS post-processed data. A normality test using Kolmogorov-Smirnov and Shapiro-Wilk revealed Gaussian distributed SNR data for all groups (p > 0.05). Therefore, a randomized block design ANOVA was used indicating significant differences among the three groups (p < 0.001). The Tukey HSD post hoc test revealed significant differences between original and POAS post-processed data (p < 0.001), original and averaged data (p < 0.001) and averaged and POAS post-processed data (p < 0.001).

### TBSS analysis of FA and MD

**Fig 3:**
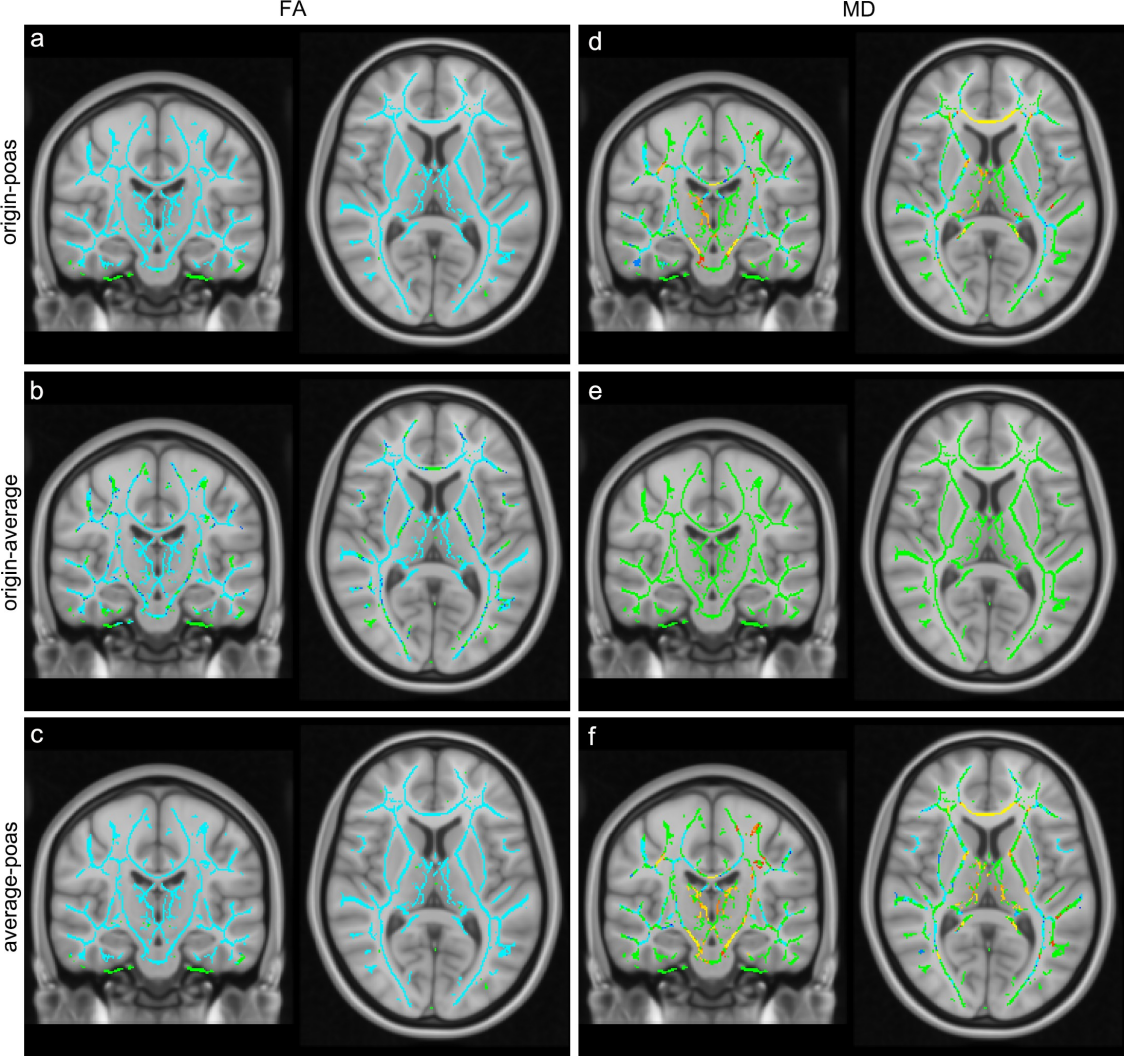
TBSS analysis among original, averaged and POAS post-processed data. Results of TBSS analysis of FA and MD among original, averaged and POAS post-processed data with fiber tract skeleton visualized in green. FA was significantly reduced (blue) in POAS post-processed data vs. original data (a), averaged data vs. original data (b) and POAS post-processed data vs. averaged data (c). MD was partially significantly reduced (blue) and partially significantly increased (yellow to red) in POAS post-processed data vs. original data (d) whereas no significant alteration of MD was seen for averaged data vs. original data (e). In POAS post-processed data vs. averaged data MD was partially significantly reduced (blue) and partially significantly increased (yellow to red) (f).

Applying TBSS, alterations in FA and MD among the three groups were analyzed within the whole brain, see Fig 3. Regarding FA, a significant decrease across the whole brain was seen in POAS post-processed data compared to original data (p < 0.05) as well as in averaged data compared to original data (p < 0.05). FA was also significantly decreased in POAS post-processed data compared to averaged data (p < 0.05) across the whole brain. Regarding MD, a significant decrease was observed in several regions such as white matter of frontal, temporal, parietal, occipital lobe and posterior limb of internal capsule as well as a significant increase in some regions such as the thalamus, corpus callosum and cerebral peduncle in POAS post-processed data compared to original data. Similar alterations were also seen in POAS post-processed data compared to averaged data with a significant decrease in regions such as white matter of frontal, temporal, parietal and occipital lobe and a significant increase in regions such as the thalamus, corpus callosum and cerebral peduncle. Comparing averaged data and original data no significant alteration was detected.

### Analysis of intra-subject variability in DWI intensity and FA

**Fig 4:**
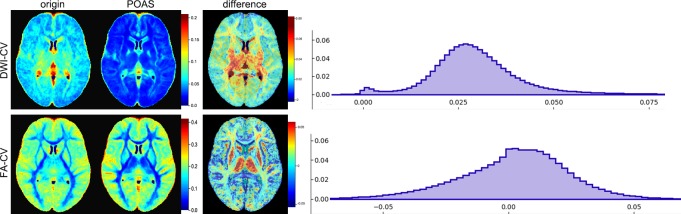
Intra-Subject variability of DWI signal intensity and FA. CV for DWI signal intensity (DWI-CV origin and POAS) and FA (FA-CV origin and POAS) as well as CV difference maps for DWI signal intensity (DWI-CV difference) and FA (FA-CV difference) showing high intra-subject variability within the white matter and central structures such as the thalamus. The histogram graphs (right) show a (moderate) horizontal shift to the right indicating (slightly) higher intra-subject variability in original data compared to POAS post-processed data.

Intra-subject variability among the repeated DWI data sets was assessed using CV maps for DWI signal intensity and FA for original and POAS post-processed data, see Fig 4 (left). Concerning DWI signal intensity mean CV was 0.072 ± 0.034 (original data) and 0.042 ± 0.031 (POAS post-processed data). Regarding FA mean CV was 0.156 ± 0.072 (original data) and 0.153 ± 0.079 (POAS post-processed data), respectively. A normality test using Kolmogorov-Smirnov and Shapiro-Wilk revealed Gaussian distributed mean CV regarding DWI signal intensities and FA (p > 0.05). Therefore, paired t-tests were applied indicating significant differences between mean CV of POAS post-processed data and original data (DWI signal intensity: p < 0.001, FA: p = 0.006). To further compare intra-subject variability across original and POAS post-processed data, CV difference maps were analyzed showing higher intra-subject variability of DWI signal intensity (mean CV difference: 0.030 ± 0.013) and FA (mean CV difference: 0.002 ± 0.022) in original data within the white matter, especially within the thalamus, see Fig 4 (middle). This was also supported by the histograms of CV difference maps showing a shift to the right for DWI signal intensity and only a mild shift to the right for FA corresponding to (slightly) higher intra-subject variability in original data in contrast to POAS post-processed data.

### Fiber tractography of the CST

**Fig 5:**
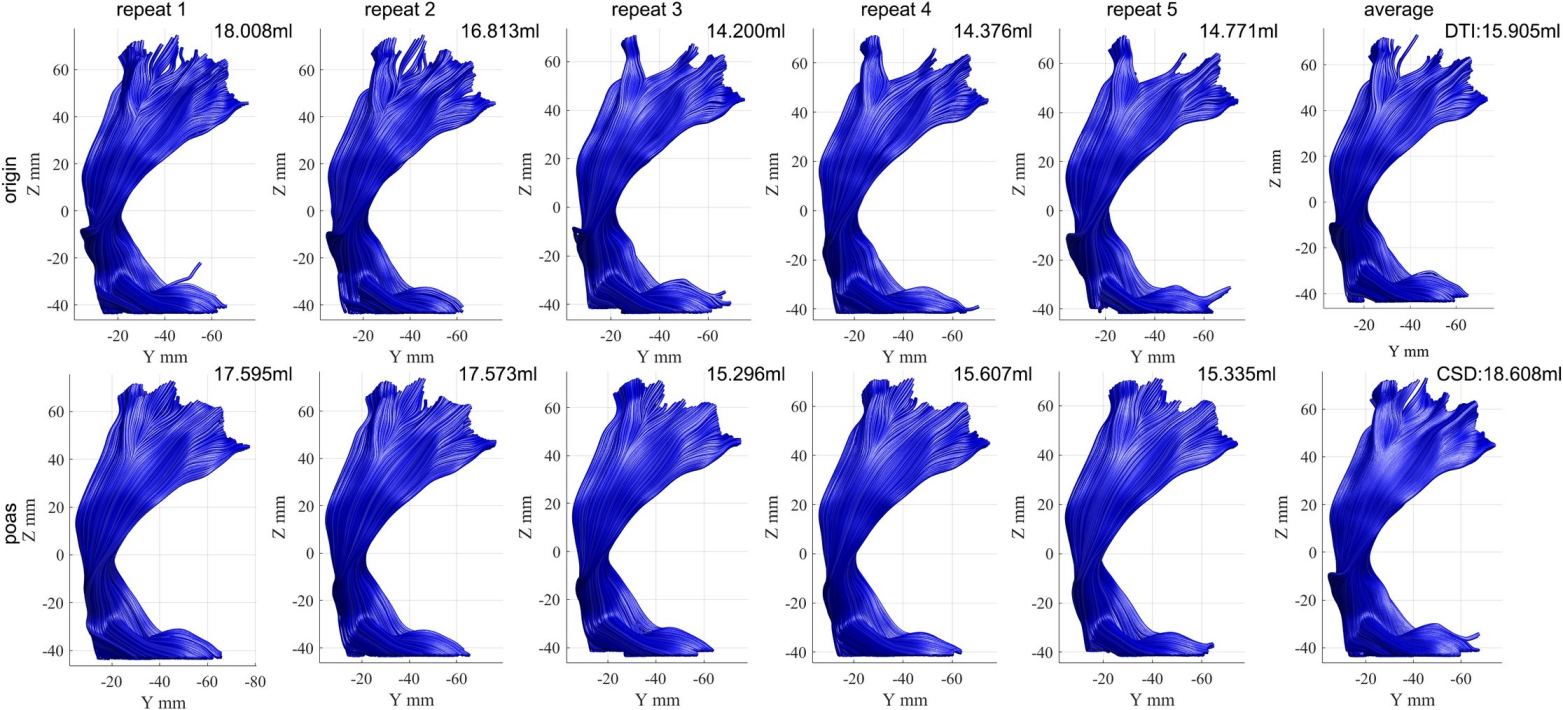
Illustrative case of fiber tractography of the CST. Exemplary CST fiber tractography results based on DWI data (volunteer 1) including tractography based on original data (upper row, column 1–5), averaged data (upper row, column 6), POAS post-processed data (bottom row, column 1–5) using the FACT algorithm as well as CSD based fiber tractography used as standard reference (bottom row, column 6).

Automated fiber tractography of the CST was performed for all original, POAS post-processed and averaged data sets using the FACT algorithms, as well as applying the CSD reconstruction on the averaged data sets as standard reference, see Fig 5. Across the five original data sets obvious differences were seen in regions above the internal capsule, especially within the motor cortex, where as in POAS post-processed data sets more consistent fiber tracking results were observed. Reconstructions based on POAS post-processed data sets additionally showed denser fiber reconstructions especially in regions close to the motor cortex in contrast to original and averaged data sets. Furthermore, results of POAS post-processed fiber tractography resemble the standard reference more than fiber tractography based on original and averaged data sets.

Tract profile analysis of fiber tractography of the CST including tract volume, FA and MD value as well as fiber density was performed, see Table 1.

**Table 1:** Results of tract profile analysis of fiber tractography of the CST.

	Side	original data	POAS data	average data	p-value* (ANOVA)	p-value# (original vs. POAS)	p-value# (original vs. average)	p-value# (POAS vs. average)
**CST volume**	L	11.32 ± 2.85	11.82 ± 2.64	11.47 ± 2.93	0.030	0.027	0.714	0.150
	R	14.37 ± 3.37	14.88 ± 3.22	14.63 ± 3.49	0.018	0.014	0.297	0.323
**FA value**	L	0.62 ± 0.02	0.59 ± 0.03	0.61 ± 0.03	< 0.001	< 0.001	0.469	< 0.001
	R	0.59 ± 0.03	0.55 ± 0.03	0.59 ± 0.03	< 0.001	< 0.001	0.092	< 0.001
**MD value**	L	0.72 ± 0.02	0.71 ± 0.02	0.72 ± 0.02	< 0.001	< 0.001	0.506	< 0.001
	R	0.73 ± 0.02	0.72 ± 0.02	0.73 ± 0.02	< 0.001	< 0.001	0.150	< 0.001
**fiber density**	L	65.50 ± 6.48	64.30 ± 6.84	65.59 ± 7.80	0.151	0.234	0.991	0.188
	R	65.91 ± 6.13	62.29 ± 5.38	66.36 ± 6.66	< 0.001	< 0.001	0.720	< 0.001

*: randomized block design ANOVA

#: Tukey HSD post-hoc test.

Across original data sets the mean tract volume was 11.32 ± 2.85 ml (left CST) and 14.37 ± 3.37 ml (right CST). CST fiber tractography based on POAS post-processed data revealed mean tract volumes of 11.82 ± 2.64 ml (left CST) and 14.88 ± 3.22 ml (right CST) whereas based on averaged data sets mean tract volume was 11.47 ± 2.93 ml (left CST) and 14.63 ± 3.49 ml (right CST). CSD fiber tractography (standard reference) resulted in mean tract volumes of 14.01 ± 3.30 ml (left CST) and 17.46 ± 4.11 ml (right CST). A normality test using Kolmogorov-Smirnov and Shapiro-Wilk revealed Gaussian distributed tract volumes for all groups (p > 0.05). Therefore, a randomized block design ANOVA was applied indicating significant difference among the all groups for the left CST (p = 0.030) and the right CST (p = 0.018). The Tukey HSD post hoc test thereby showed significant differences between fiber tractography based on original and POAS post-processed data (left CST: p = 0.027, right CST: p = 0.014). Comparing POAS post-processed and averaged data (left CST: p = 0.150, right CST: p = 0.323) and original and averaged data (left CST: p = 0.714, right CST: p = 0. 297), no significant differences in tract volume were found.

Analyzing FA along fiber tractography results of the CST, mean FA value was 0.616 ± 0.024 (left CST) and 0.593 ± 0.026 (right CST) across original data sets. For POAS post-processed data mean FA value was 0.568 ± 0.027 (left CST) and 0.547 ± 0.030 (right CST), whereas for averaged data mean FA was 0.613 ± 0.025 (left CST) and 0.588 ± 0.027 (right CST) within the CST reconstruction. A normality test using Kolmogorov-Smirnov and Shapiro-Wilk showed Gaussian distributed mean FA values for all groups (p > 0.05). Therefore, a randomized block design ANOVA was applied revealing significant difference among the all groups for left CST and right CST (p < 0.001). Tukey HSD post hoc tests showed significant differences in mean FA value within the CST between original and POAS post-processed as well as POAS post-processed and averaged data (p < 0.001). However, no significant difference was seen between original and averaged data regarding mean FA value (left CST: p = 0.469, right CST: p = 0.092). Along the CST a mean MD value of 0.723 ± 0.020 (left CST) and 0.729 ± 0.020 (right CST) across original data sets, 0.714 ± 0.019 (left CST) and 0.720 ± 0.020 (right CST) for POAS post-processed data sets and 0.722 ± 0.020 (left CST) and 0.727 ± 0.020 (right CST) for averaged data sets. A normality test using Kolmogorov-Smirnov and Shapiro-Wilk showed Gaussian distributed mean MD values for all groups (p > 0.05). Therefore, a randomized block design ANOVA was applied revealing significant difference among the all groups for left CST and right CST (p < 0.001). Tukey HSD post hoc tests showed significant differences in mean MD value within the CST between original and POAS post-processed as well as POAS post-processed and averaged data (p < 0.001). However, no significant difference was seen between original and averaged data regarding mean MD value (left CST: p = 0.506, right CST: p = 0.150).

Finally, fiber density was 65.50 ± 6.483 (left CST) and 65.91 ± 6.132 (right CST) for original data sets, 64.30 ± 6.838 (left CST) and 62.29 ± 5.382 (right CST) for POAS post-processed data as well as 65.59 ± 7.800 (left CST) and 66.36 ± 6.654 (right CST) for averaged data. A normality test using Kolmogorov-Smirnov and Shapiro-Wilk showed Gaussian distributed fiber density for all groups (p > 0.05). Therefore, a randomized block design ANOVA was applied and a significant difference among all groups was seen only for the right CST (p < 0.001). Tukey HSD post hoc test shows significant differences for the right CST between original and POAS post-processed as well as POAS post-processed and averaged data (p < 0.001). However, no significant difference was seen between original and averaged data (p = 0.720).

### Reproducibility and similarity analysis of the CST

Calculation of the JC across the five original data sets revealed JCs of 0.313 ± 0.048 (left CST) and 0.372 ± 0.059 (right CST), whereas across POAS post-processed data JCs of 0.408 ± 0.071 (left CST) and 0.457 ± 0.055 (right CST). A normality test using Kolmogorov-Smirnov and Shapiro-Wilk showed Gaussian distributed JC values (p > 0.05). Therefore, a paired t-test resulted in a statistical significant difference between reproducibility assessed by the JC between original and POAS post-processed data for left and right CST (p < 0.001).

The JC was also used to estimate the similarity of CST fiber tractography between original and POAS post-processed data which was 0.625 ± 0.045 for the left CST and 0.657 ± 0.051 for the right CST. To further compare CST fiber tractography using original and POAS post-processed data the similarity with the standard reference of the CST conducted using CSD tractography was assessed. JC between fiber tractography using original data and the standard reference was 0.641 ± 0.057 (left CST) and 0.687 ± 0.053 (right CST), whereas JC between fiber tractography using POAS post-processed data and the standard reference was 0.599 ± 0.054 (left CST) and 0.643 ± 0.053 (right CST). A normality test using Kolmogorov-Smirnov and Shapiro-Wilk showed no Gaussian distribution of JC for all groups (p < 0.05). Therefore, the Wilcoxon signed-rank test revealed significant differences between the similarity of fiber tractography of original data and standard reference and fiber tractography of POAS post-processed data and standard reference for left and right CST (p < 0.001).

## Discussion

In this study we analyzed the contribution of POAS post-processing of DWI data regarding image quality and SNR, diffusion tensor metrics and fiber tractography of the CST in comparison to original data and averaged data, as the standard approach to increase image quality. Averaging as standard approach to increase image quality is accompanied with blurring, also alleviating anisotropic structures. In contrast, POAS post-processing is an edge-preserving denoising approach obtaining fine and anisotropic structures and therefore avoids blurring of inherent structures and preserves discontinuities [46, 47].

Comparing the SNR across original data, averaged data and POAS post-processed data, POAS post-processing was capable of significantly increasing SNR (especially reducing noise) compared to original data, but also averaged data without blurring inherent structures or boundaries between white and grey matter (see Fig 2). Notably, image quality was not only quantitatively but also qualitatively improved applying POAS post-processing, especially in comparison to the standard approach of averaging. Considering the potential conflict between spatial resolution, SNR and acquisition time, POAS post-processing might be an applicable approach to increase SNR while increasing spatial resolution without time-consuming repeated acquisitions to compensate for the decreased SNR according to higher spatial resolution.

POAS post-processing appears suitable to reduce noise and enhance image quality. Especially noise in DWI introduces systematically effects, also in diffusion metrics such as FA and MD [68, 69]. Although many previous studies analyzed denoising algorithms in DWI data only a few articles investigated the influence of these denoising strategies on diffusion metrics. To evaluate whether FA and MD values were affected by denoising using POAS post-processing and averaging, a TBSS analysis was conducted. A significant decrease in FA across the whole brain was seen applying averaging (Fig 3B) which is consistent with previous studies. Those reported that increased SNR was accompanied with decreased FA while lower SNR led to an overestimation of FA to some degree [27, 70–72]. Applying POAS post-processing FA was decreased across the whole brain in comparison to original data but also to averaged data (Fig 3A and 3C), which could in part be explained by the considerable improvement in SNR of POAS post-processed data in line with the previous mentioned study results. As SNR is also increased in averaged data, but without reduction of FA, the application of POAS might contribute in an additional way by smoothing the sphere of diffusion gradients or more isotropic tensors due to smoothing. However, even though FA was also decreased in contrast to averaging, it still remains unclear whether averaging or POAS post-processing led to FA values closer to the real FA value although other similar denoising methods had already proven to shift FA estimations closer to real FA values within simulated data [37]. POAS post-processing also led to lower intra-subject variability in FA in contrast to original data that could also be explained by lower intra-subject variability in DWI signal intensity, less noise and higher SNR in DWI data which might also contribute to decreased FA in POAS post-processed data.

Especially in clinical applications in neurosurgery, an accurate and reliable visualization of major white matter tracts such as the CST is required to minimize the risk of postoperative neurological deficits [9–11]. Therefore, particularly the effect of POAS post-processing on fiber tractography of the CST and on tract profile characteristics is of special interest. Even though mean FA was significantly reduced in POAS post-processing, tract volume was larger in contrast to original data, whereas standard averaging did not significantly increase the tract volume. Furthermore, CST tractography results in POAS post-processed data revealed smoother, anatomically more accurate reconstructions, mostly comparable to the reconstructed standard reference in comparison to tractography based on original or averaged data (see Fig 5). The standard reference was created using CSD based on a tensor free model with an approach to characterize intra-voxel diffusion behavior [64, 65]. CSD-based tractography enables the reconstruction of more complex fiber architectures compared to the single diffusion tensor model of DT based approaches, especially in pathological cases [63]. Therefore, the high comparability of tractography based on POAS post-processed data and the standard reference might lead to the conclusion, that POAS post-processing enables even DTI-based approaches to better reflect the complexity of fibers and the anatomical structure of the CST.

Reproducibility and similarity between CST reconstructions based on original and POAS post-processed data was analyzed utilizing the JC. The JC was significantly higher in case for POAS post-processed data revealing higher reproducibility and lower variability among the reconstruction of the CST in contrast to those of the original data, which is also one aim of higher angular resolution data besides resolving the complexity of fiber structures [20, 73]. Fiber tractography based on HARDI, by adding further diffusion encoding gradients and increasing the b-value, delivers improved reconstructions also of crossing and intermixing fibers as well as further reproducible and stable results, both shortcomings of the standard DTI model [18, 23, 24]. The results of this study show high reproducibility among several data sets and higher visual similarity to the standard reference compared to original and even averaged data. Thereby POAS post-processing seems to compensate at least partially for the drawbacks of the standard DWI data acquisition and standard DTI application leading to further accurate and reproducible fiber tractography results.

Inspecting the quantitative similarity metrics between standard reference and CST reconstructions based on original and POAS post-processed data assessed by the JC, a significantly lower similarity was found for POAS post-processed data even though significantly larger tract volumes were seen for POAS post-processed data while FA was significantly decreased across the brain, especially along the CST. As the CST is a major white matter tract with high fiber integrity compared to other white matter tracts such as the optic radiation, a decrease in FA might not lead to an alongside decrease in CST volume [74], but rather to an increased one. However, lower FA might be related to lower fiber density [75], which was also seen in our study results. The reconstructions of the CST based on POAS post-processed data showed lower fiber density, in combination with decreased FA in CST. Probably the lower fiber density within the CST is based on POAS post-processing contributing to the lower similarity between POAS post-processed data and the standard reference.

Besides POAS, many other denoising approaches were reported to improve image quality, such as Gaussian filtering [30], wavelet transformation[31], non-linear anisotropic diffusion filter [33], the propagation-separation approach [34], non-local means [35], Perona-Malik filter [32], linear minimum mean square error estimation [36], local principal component analysis [37], random matrix theory [38], higher-order singular value decomposition [39] and advanced adaptations of non-local means based approaches [40–43] or machine learning methods [44]. In this study, POAS post-processing led to improved SNR enhancement while avoiding edge-blurring which is essential not only for fiber tractography. Averaging is a common approach to increase SNR by enhancing the signal using multiple acquisitions [72]. It is known to suppress the effects of random variations or random artefacts and was reported to reduce physiological artefacts, such as like respiratory motion [29]. However, this approach is obviously accompanied with an increased acquisition time being a major drawback in the clinical application. POAS post-processing, on the contrary, makes use of a single acquisition, clinically feasible, with even a further improvement in SNR and fiber tractography of the CST regarding shape and complexity with comparable tract volumes. Although post-processing using POAS is a time-consuming additional step and up to now might thereof be of limited use for immediate intraoperative applications when using intraoperative MRI, in contrast to other approaches, the acquisition time for DWI data is not affected, making it applicable within the clinical routine. Especially in clinical applications where high spatial and angular resolution might be limited due to time consuming acquisition (increased resolution, averaging, etc.), POAS post-processing might support the gain of high quality data. Thereby for example using a given time-frame for DWI acquisition POAS post-processing could be used to acquire data with higher resolution (spatial, angular) instead of repeating measures to increase quality of data with low spatial and angular resolution.

Within the past decades DTI and fiber tractography became a common and feasible method for a wide range of clinical and neuroscience applications. Even though DTI is well established and evaluated it is known that DTI is not capable of resolving crossing and kissing fibers or multi-fiber populations [76]. Therefore, further advanced and more complex diffusion models or model free approaches are needed to overcome those limitations. So far several approaches have been published, most of them making use of higher angular resolution and/or multiple b-values such as Q-Ball imaging [25] or diffusion spectrum imaging [77]. Despite limitations of the routinely used second-order tensor model, the application of POAS post-processing seems could thereby be helpful to keep acquisition times short while achieving high image quality also feasible for clinical applications. Improving image quality and fiber tractography, spatial and angular resolution are important factors. Thereby, higher angular resolution seems to be more important regarding the improvement of fiber tractography also within clinical applications with limited acquisition times [19]. Therefore, POAS post-processing might be a useful and clinically applicable contribution to improve stability and complexity in fiber tractography of the CST by enhancing image quality (regarding e.g. the SNR), with no increase in acquisition time. Further investigations are needed to evaluate its potential in clinical applications, especially in pathological cases. With improvements in image acquisitions techniques regarding shortening acquisition times (e.g. using multi echo imaging) also multi-shell DWI will be applicable in clinical routine, where POAS or especially its advancement for multi-shell data (multi-shell POAS, [47]) will be further investigated.

Nonetheless, there are also some limitations. Besides increased post-processing times, after denoising applying POAS post-processing FA significantly decreased across the whole brain as well as fiber density along the CST. Even if in neurosurgical applications, the spatial extent of white matter tract reconstructions is of greater importance than fiber density of diffusion metrics, there might be other applications such as neurological, psychiatric or neuroscientific applications, where tract metrics are of greater interest possibly reflecting dynamic changes of microstructure in diseases. In these cases, POAS post-processing should also be further investigated focusing on tract metrics. Data averaging is a common and simple approach to increase image quality and available in most commercial and research image processing platforms. In case of DWI data recent studies such as [40] have shown that additional filtering to overcome bias due to Rician noise as present in DWI data for example accounts for higher quality averaged data and thereby seem to lead to improved reference data for comparison of new denoising algorithms.

## Conclusions

In this study, POAS post-processing was investigated in comparison to original data and averaged data being the standard approach to improve image quality and associated metrics. POAS post-processing was capable of reducing noise and improving SNR of DWI data significantly and reduced the bias and intra-subject variability of diffusion metrics such as FA. Fiber tractography resulted in larger tract volumes and more accurate and reliable reconstructions of complex fiber architecture of the CST with high reproducibility and less variability among different data sets. Therefore, POAS post-processing affects different levels of DWI data processing (image quality, diffusion metrics, fiber tractography) and seems to partly compensate for the drawbacks of spatial and angular resolution. Further work will include its application in pathological cases with altered microstructure to conduct and verify its value in the clinical, especially neurosurgical application.
